# Response of soil fungal communities to continuous cropping of flue-cured tobacco

**DOI:** 10.1038/s41598-020-77044-8

**Published:** 2020-11-16

**Authors:** Shengnan Wang, Jiangke Cheng, Tong Li, Yuncheng Liao

**Affiliations:** 1 College of Agronomy, Northwest A&F University, Yangling, 712100 Shaanxi China; 2 School of Biological and Chemical Engineering, Panzhihua University, Panzhihua, 617000 Sichuan China; 3 School of Mathematics and Computer Science, Panzhihua University, Panzhihua, 617000 Sichuan China

**Keywords:** Ecology, Microbiology, Plant sciences

## Abstract

Fungal communities are considered to be critically important for crop health and soil fertility. However, our knowledge of the response of fungal community structure to the continuous cropping of flue-cured tobacco is limited, and the interaction of soil fungal communities under different cropping systems remains unclear. In this study, we comparatively investigated the fungal abundance, diversity, and community composition in the soils in which continuous cropping of flue-cured tobacco for 3 years (3ys), 5 years (5ys), and cropping for 1 year (CK) using quantitative polymerase chain reaction and high-throughput sequencing technology. The results revealed that continuous cropping of flue-cured tobacco changed the abundance of soil fungi, and caused a significant variation in fungal diversity. In particular, continuous cropping increased the relative abundance of Mortierellales, which can dissolve mineral phosphorus in soil. Unfortunately, continuous cropping also increased the risk of potential pathogens. Moreover, long-term continuous cropping had more complex and stabilize network. This study also indicated that available potassium and available phosphorous were the primary soil factors shifting the fungal community structure. These results suggested that several soil variables may affect fungal community structure. The continuous cropping of flue-cured tobacco significantly increased the abundance and diversity of soil fungal communities.

## Introduction

Flue-cured tobacco is a crop intolerant to continuous cropping, but because of limited cultivation land and lack of a sustainable cropping concept, the majority of tobacco in mountainous areas of southwest China is usually cropped in continuous cropping system. This can lead to the loss of soil nutrients and imbalance of nitrogen, phosphorus and potassium in the soil^1^. It is known that soil enzyme activity decreases significantly after repeated cropping, resulting in a decline of tobacco yield and quality declined^2^. Continuous cropping has seriously affected sustainable development of flue-cured tobacco production.

Soil is the basis for sustainable development of the environment, and it provides the matrix and nutrients necessary for crop growth. As the key component of farmland ecosystems, soil microorganisms promote the decomposition of soil organic matter and transformation of soil nutrients. They play an important role in maintaining soil quality and health. The soil microbial community structure can be used as the earliest observable soil quality index in the terrestrial ecosystems. Continuous cropping of flue-cured tobacco can change the soil nutrient content and enzyme activity, causing the flue-cured tobacco roots to secrete chemicals which lead to self-toxicity^3^. All of this has a significant influence on the diversity of soil microbial community structure. Continuous cropping of flue-cured tobacco changes soil microbial biomass and diversity, and it leads to the occurrence of continuous cropping diseases, which is not conducive to the development of soil ecosystems.

Fungi play a major role in the soil ecosystems. They are involved in soil nutrientcirculation, organic matter decomposition, and crop pathology, and they are closely linked to crop health and growth^4,5^. Certain fungus in the soil were plant pathogens that cause plant diseases, whereas some others were biocontrol factors that can inhibit or decrease the impact of plant diseases^6^. Soil fungal community structure is obvious impacts by the soil physico-chemical properties^7^. Soil pH and enzyme activity are strongly associated with rhizosphere fungal communities as well^8^. Thus, improving the knowledge on soil fungal communities should increase our understanding of their roles in soil ecosystems.

In agroecosystems, agricultural management practices significantly influenced the soil fungal communities^9^. In continuous cropping systems, constant root secretions from the same crop species may increase the abundance of plant pathogens and aggravate soil–borne fungal diseases, which may negatively affect these agroecosystems. The studies have shown that continuous cropping of eggplant^10^, cotton^11^ and soybeans^12^ increased number of the pathogens. However, most studies on microbial diversity under continuous cropping of flue-cured tobacco focused on the influence of microbial quantity and bacterial community structure in soil. Studies on the effect of continuous cropping of flue-cured tobacco on soil fungal community structure are limited.

In present study, we collected soil samples from three different cultivation sites: flue-cured tobacco cropping for 1 year, flue-cured tobacco continuously cropped for 3 years, and tobacco continuously cropped for 5 years. We compared the abundance, diversity, and composition of their fungal communities using quantitative polymerase chain reaction (qPCR) and high-throughput sequencing. We hypothesized that the different continuous cropping treatments would lead to the selection of distinct soil fungal communities as a consequence of their different soil physico-chemical properties. Consequently, we had two main objectives: (1) to determine the effects of continuous cropping of flue-cured tobacco on on both fungal communities and soil properties; (2) to examine the correlations between soil properties and soil fungal communities under continuous cropping systems.

## Results

### Soil physico-chemical properties and enzyme activity

The physico-chemical properties and enzyme activity of the soil samples in different cropping systems of flue-cured tobacco are shown in Table 1. The edaphic physico-chemical properties differed significantly between the three treatments. The content of SOC and NO^3−^-N under continuous cropping (3ys and 5ys) were significantly lower than those in CK. The soil Ava-P accumulates with the increase of cropping years, 5ys was highest with 31.656 mg kg^−1^. However, the content of Ava-K decreased with the increase of croppinging years, the highest was recorded in CK, with 186.752 mg kg^−1^, and the lowest was 133.041 mg kg^−1^ in 5ys.

**Table 1:** Soil properties according to the different cropping systems of flue-cured tobacoo.

	pH	SOC (g kg^−1^)	TN (g kg^−1^)	NO^3−^-N (mg kg^−1^)	NH^4+^-N (mg kg^−1^)	Ava-P (mg kg^−1^)	Ava-K (mg kg^−1^)	Sucrase (mg kg^−1^)	Urease (mg kg^−1^)	Catalase (mg kg^−1^)
3 yr	6.02 b	16.024 b	1.64 b	12.200 b	22.210	13.169 b	185.241 a	8.233	1.176 b	1.584 a
5 yr	6.13 a	15.609 b	2.42 a	14.590 b	29.980	31.656 a	133.041 b	8.03	1.019 b	1.287 b
CK	6.20 a	29.802 a	2.216 a	22.110 a	20.730	11.638 b	186.752 a	6.673	2.571 a	1.652 a
*D.F*	8	8	8	8	8	8	8	8	8	8
*P* value	0.01**	0.01**	0.01**	0.02**	*ns*	0.01**	0.01**	*ns*	0.01**	0.042**

Different letters and asterisk indicated significant differences among soil samples as calculated by One-way ANOVA and *t* test. Two asterisks indicated significant differences at the *P* < 0.01 level. SOC: soil organic carbon, TN: total nitrogen, NO^3–^-N: nitrate-nitrogen, NH^4^^+^-N: ammonium-nitrogen, Ava-P: available phosphorus, Ava-K: available potassium, *ns*: not significant (*P* > 0.05). 3ys: continuous cropping of flue-cured tobacco for 3 years. 5ys: continuous cropping of flue-cured tobacco for 5 years. CK: flue-cured tobacco cropping for 1 year. *D.F.* : degree of freedom.

We also found that continuous cropping significantly decreased the activities of Urease and Catalase in the soil, and their contents were the lowest in 5 yr (1.019 mg kg^−1^ and 1.287 mg kg^−1^, respectively).

### Fungal abundance and alpha diversity in the soil

**Figure 1:**
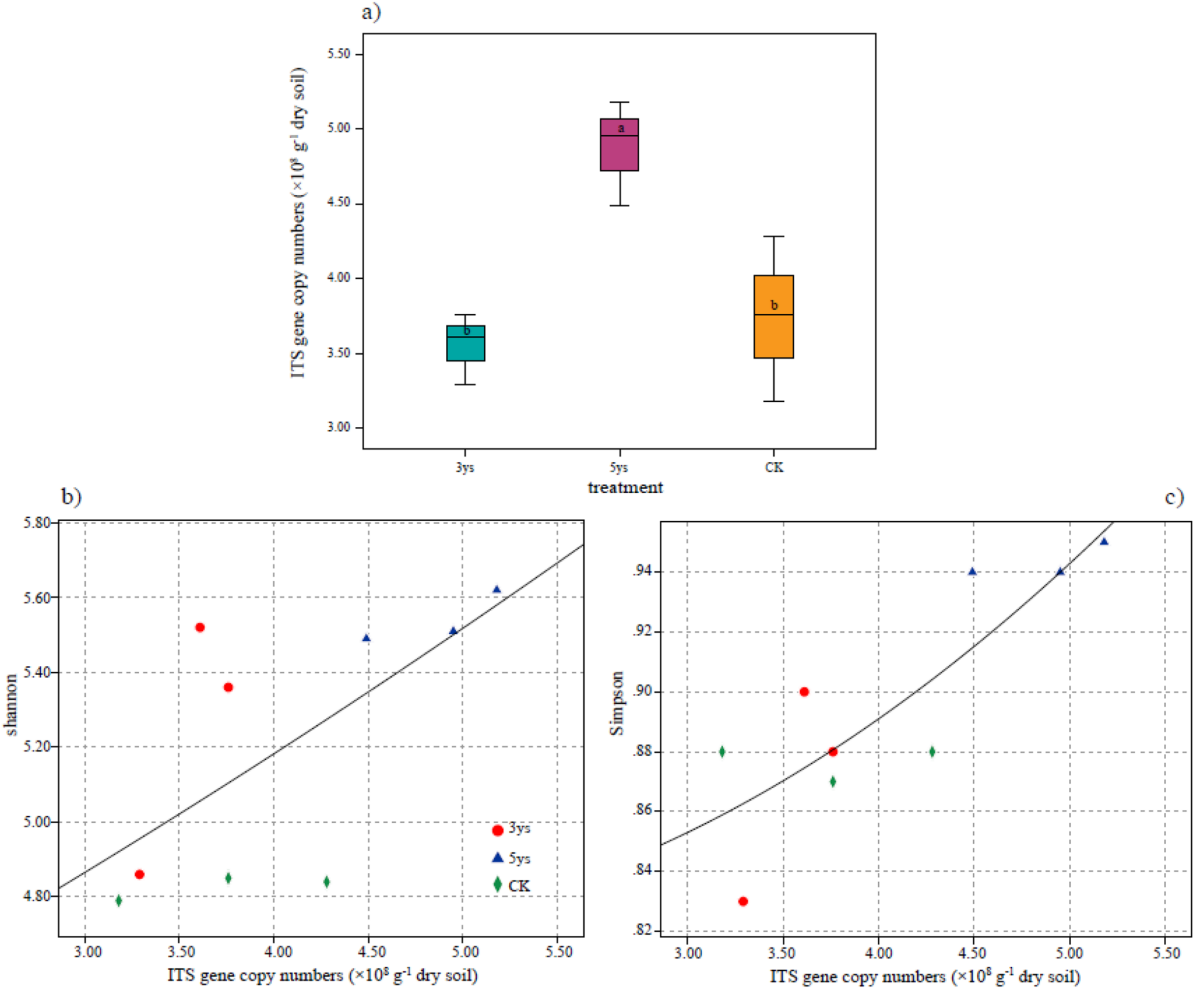
Fungal abundance of fungal ITS gene (**a**), and the regression model of the relationship between the fungal abundance and (**b**) Shannon’ diversity index (H’ value), (**c**) Simpson’s diversity index. Different letters indicated significant differences among soil samples as calculated by One-way ANOVA and *t* test, (*P* < 0.01). 3ys: continuous cropping of flue-cured tobacco for 3 years. 5ys: continuous cropping of flue-cured tobacco for 5 years. CK: flue-cured tobacco cropping for 1 year.

The abundance of fungi in different soils under different cropping systems ware significantly different (Fig. 1a). The abundance of fungi increased with the increase of the cropping years, and the 5ys had the highest fungal abundance of 4.87 × 10^8^ g^−1^ dry soils. Statistically significant differences in soil fungal diversity under different cropping systems were obtained using the Shannon’s H value and Simpson indices. (Fig. 1b,c). 5ys had the highest Shannon’s H value of 5.54, followed by 3ys, and the CK had the lowest Shannon’s H value of 4.83. Similarly, 5ys has the highest Simpson’s index of 0.94. Both Shannon and Simpson indices showed a significant correlation between community abundance and diversity indices. The diversity of fungi in soil under continuous cropping was higher than that cropping for 1 year. Interestingly, the abundance and diversity of the fungal communities increased with the increase in the duration of continuous cropping. Fungal abundance and diversity in 5ys were higher than those in 3ys and CK.

### Fungal community composition in the soil

**Figure 2:**
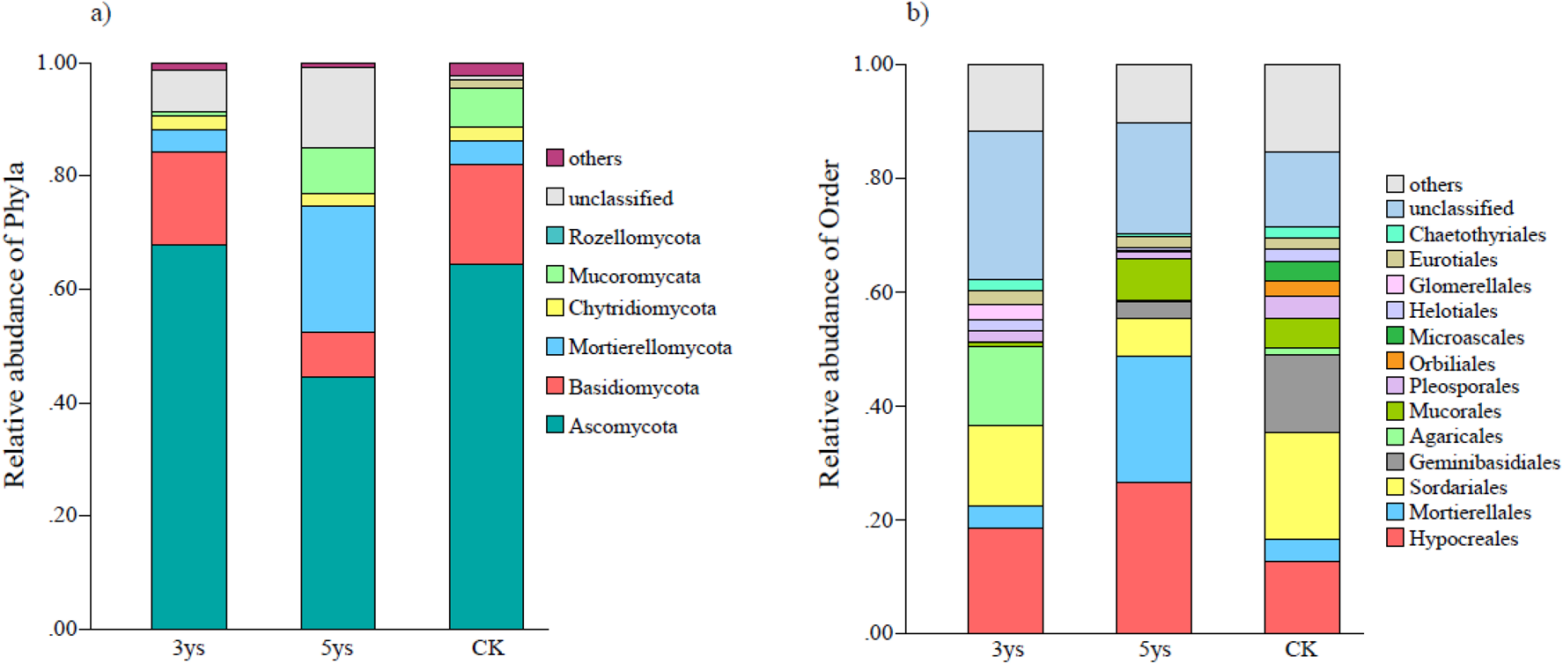
The composition of the soil fungal communities at the phylum (**a**), and order (**b**) level in the three treatments. 3ys: continuous cropping of flue-cured tobacco for 3 years. 5ys: continuous cropping of flue-cured tobacco for 5 years. CK: flue-cured tobacco cropping for 1 year.

The analysis of the soil samples yielded 312,757 quality sequences in total and 32,728–38,629 sequences per sample (mean = 34,751), and 97.52% of the sequences were classified at the phylum level. In all samples, the dominant fungal phyla were Ascomycota, Basidiomycota and Mortierellomycota, accounting for 74.70% to 88.21% of all sequences (Fig. 2a). Under different cropping systems, the relative abundances of dominant phyla were varied. In addition, Chytridiomycota, Mucoromycota, and Rozellomycota were recorded in low abundance in all the samples. The relative abundance of Ascomycota was not significantly different between CK and 3ys. However, the relative abundance of Ascomycota gradually decreased with the increase in continuous cropping duration, and in 5ys soil, it decreased by 19.93%, compared with that in CK. The relative abundance of Basidiomycota also decreased with the increase in continuous cropping duration 17.58% in CK, 16.44% in 3ys, and only 7.86% in 5ys. Interestingly, the relative abundance of Mortierellomycota had an upward trend, and in 5ys, it was 5.58- and 5.73- fold higher than that in the CK and 3ys, respectively.

The relative abundance of fungi in different cropping systems at the order level was significantly different (Fig. 2b). Sordariales was the dominant order in CK, accounting for 18.84% of all fungi, which was higher than that in 3ys (13.39%) and 5ys (6.56%). In 3ys, Hypocreales and Agaricales were the dominant orders, accounting for 18.62% and 13.94%. Hypocreales and Mortierellales were the dominant orders in 5ys, accounting for 26.57% and 22.07% of the relative abundance of all fungi, respectively.

### Soil fungal beta diversity

**Figure 3:**
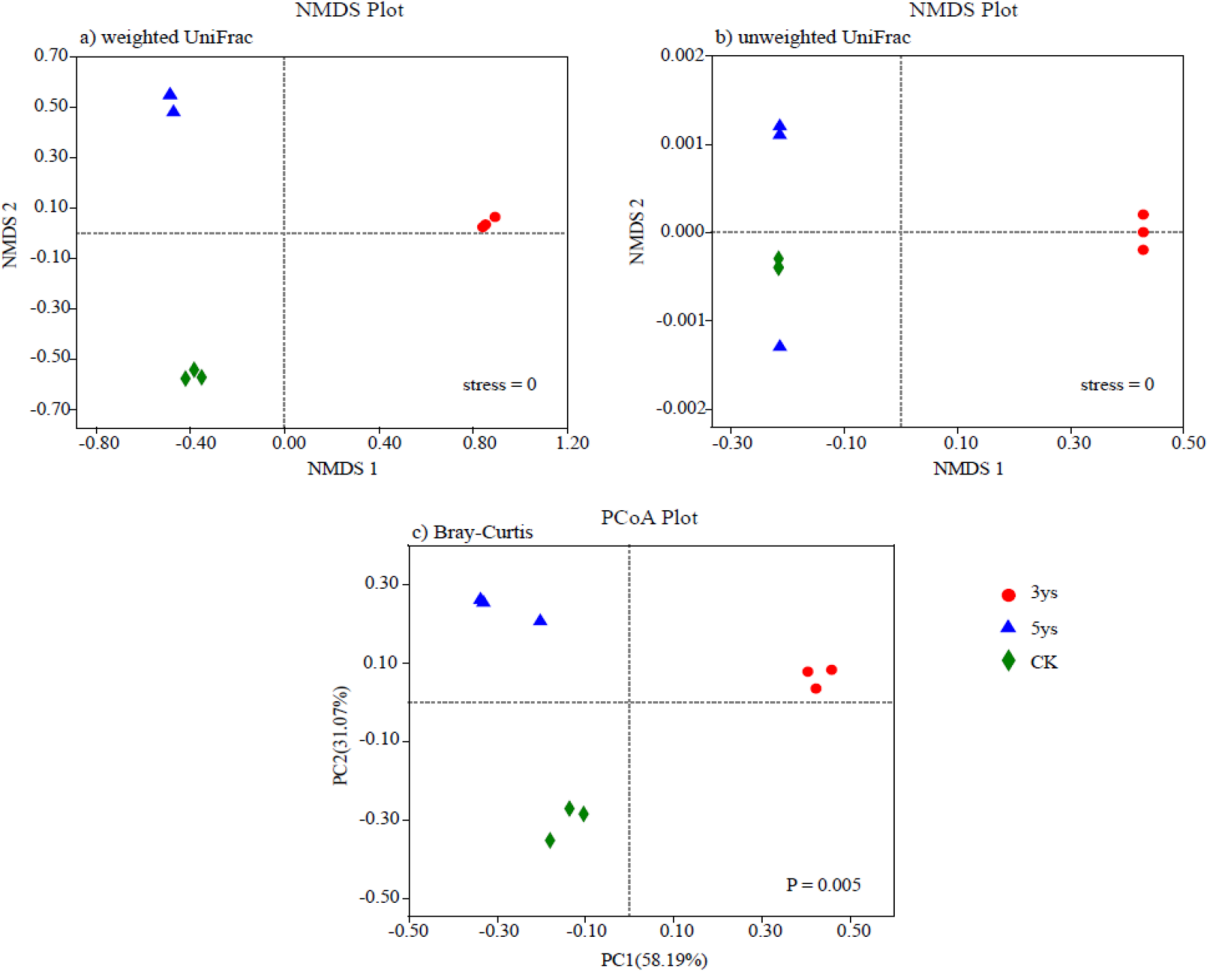
Fungal community structure indicated by non-metric multi-dimensional scaling (NMDS) plots of weighted (**a**), and unweighted (**b**) pairwise UniFrac distances. Principal coordinates analysis (PCoA) plot depicted the Bray–Curtis distance (**c**) of fungal communities. 3ys: continuous cropping of flue-cured tobacco for 3 years. 5ys: continuous cropping of flue-cured tobacco for 5 years. CK: flue-cured tobacco cropping for 1 year.

Phylogenetic analysis of community membership and composition was performed using weighted and unweighted UniFrac distances, and the changes in the fungal phylogenetic structure as a consequence of to the different cropping systems were evaluated by non-metric multidimensional scaling (NMDS) analyses (Fig. 3a,b). NMDS analyses showed that the phylogenetic structure of soil fungal communities differed under different cropping systems. The NMDS plots showed that the fungal community of each treatment was obviously separated from the others. Fungal community structure of soil under different cropping years was significantly different. Principal coordinate analysis (PCoA) data showed that the three treatments were obviously separated, and each one was far away from the other two (Fig. 3c).

**Figure 4:**
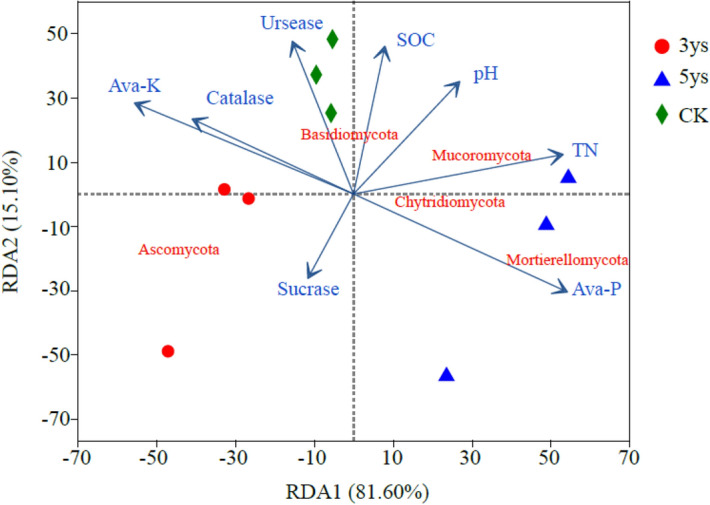
Redundancy analysis (RDA) demonstrating the relationships between soil properties and fungal community structures. The red font represents the fungal phyla with top 5 abundance; the blue arrows represent different soil properties. SOC: soil organic carbon, TN: total nitrogen, Ava-P: available phosphorus, Ava-K: available potassium. 3ys: continuous cropping of flue-cured tobacco for 3 years. 5ys: continuous cropping of flue-cured tobacco for 5 years. CK: flue-cured tobacco cropping for 1 year.

The relationships between soil properties and fungal community composition were significantly different among the investigated cropping systems (Table 2). TN (*r* = 0.532, *P* = 0.023), Ava-P (*r* = 0.752, *P* = 0.011), Ava-K (*r* = 0.780, *P* = 0.011) and Catalase activity (*r* = 0.411, *P* = 0.025) were the main factors determining the variation of fungal phylogenetic structure. In addition, redundancy analysis (RDA) showed that Ava-K made the greatest influnce on fungal community structure, and the Ava-P also had a significant influnce in shaping the fungal community structure (Fig. 4).

**Table 2:** Mantel test result for the correlation between fungal beta diversity and soil properties.

	pH	SOC	TN	NO^3−^-N	NH^4+^-N	Ava-P	Ava-K	Sucrase	Urease	Catalase
R	0.231	0.129	0.532*	− 0.045	0.347	0.752*	0.780*	− 0.170	0.026	0.411*
*P* value	0.147	0.345	0.023	0.808	0.061	0.011	0.011	0.294	0.882	0.025

SOC: soil organic carbon, TN: total nitrogen, NO^3−^-N: nitrate-nitrogen, NH^4+^-N: ammonium-nitrogen, Ava-P: available phosphorus, Ava-K: available potassium. One asterisk indicated significant differences at the 0.01 < *P* value < 0.05.

### Co-occurrence network analysis

**Figure 5:**
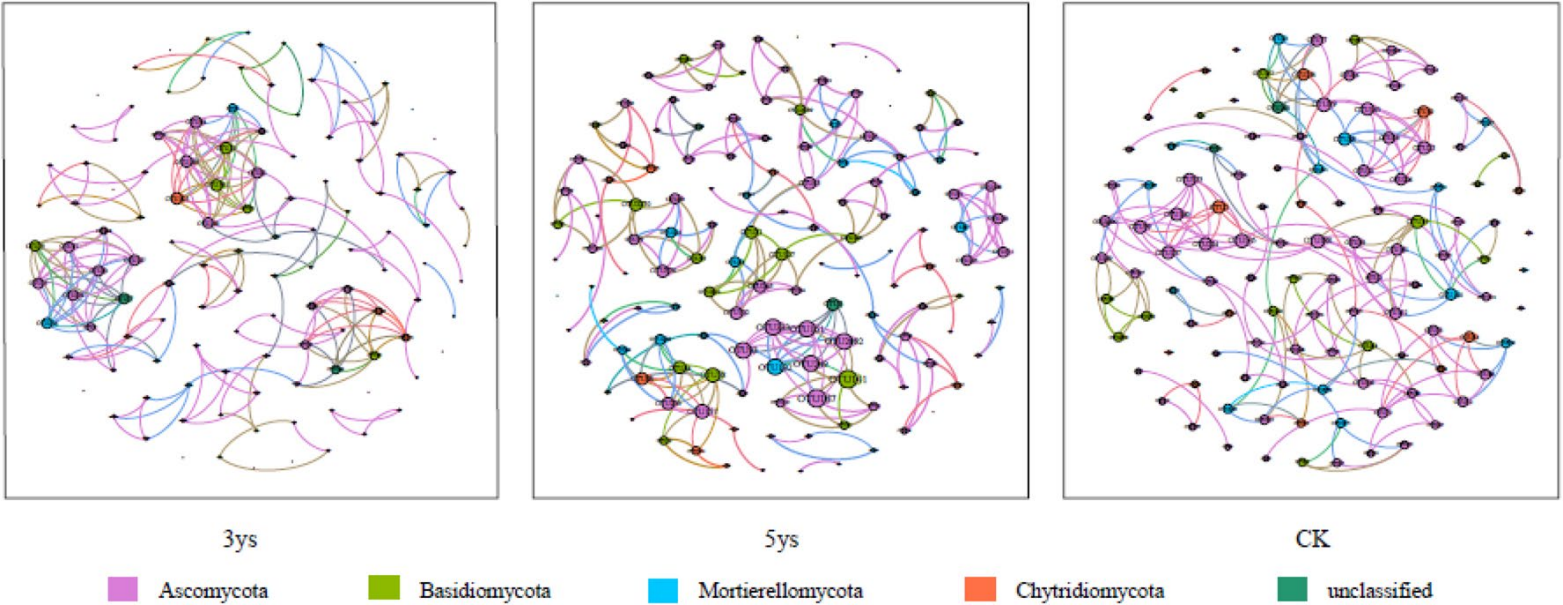
The co-occurrence network of soil fungi based correlation analysis. A connection stands for a strong (Spearman’s *ρ* > 0.6) and significant (*P* < 0.01) correlation. The nodes represent unique OTUs. The size of each node is proportional to degree. The nodes colored by taxonomy. 3ys: continuous cropping of flue-cured tobacco for 3 years. 5ys: continuous cropping of flue-cured tobacco for 5 years. CK: flue-cured tobacco cropping for 1 year.

Differences and interactions in fungal co-occurrence patterns under different cropping systems were further revealed by co-occurrence network at the OTU level (Fig. 5). The topological properties of the soil fungal network were significantly different among 3ys, 5ys and CK (Table 3). The 5ys soil fungal network had the highest number of nodes and edges, highest average degree, and greatest average path lengths among all treatments, indicating its highest stability and complexity. The OTUs with the highest betweenness centrality scores were considered as keystone taxa. In this study, OTU65 (*Cylindrocarpon *sp.) and OTU246 (*Geminibasidium *sp*.*) were categorized as keystone taxa under continuous cropping.

**Table 3:** Topological properties of fungal communities in different cropping systems.

Treatment	Nodes	Edges	Average degree	Average clustering coefficient	Average path length	Modularity
3ys	120	336	5.600	0.880	1.784	0.876
5ys	136	377	5.800	0.851	2.317	0.896
CK	119	331	5.563	0.834	2.198	0.872

3ys: continuous cropping of flue-cured tobacco for 3 years. 5ys: continuous cropping of flue-cured tobacco for 5 years. CK: flue-cured tobacco cropping for 1 year.

## Discussion

Continuous cropping of tobacco leads to changes in soil properties, nutrient imbalance, and crop yield, as well as crop quality reduction^13^. In the present study, soil TN and Ava-P in 5ys treatment were highest than those two other treatments (Table 1). This indicated that certain soil nutrients increased after long-term continuous cropping. The finding is in accordance with previous report, which state that the soil physico-chemical properties increased significantly after 13 years of soybeans monoculture^14^. Zhong et al.^15^ observed that, after continuously cropped banana, the soil available nutrients were significantly increased. Manici et al.^16^ indicated that the main reason for the increase of soil nutrient contents was that long-term continuous cropping led to an enhancement in the number of microorganisms in soil material circulation. Interestingly, our results showed that Ava-K was significantly lower in 5ys treatment than that in 3ys and CK, indicating that the content of Ava-K decreased with the increase in continuous cropping duration. The reason for this may be that flue-cured tobacco is the potassium-type crop which requires large amounts of Ava-K for growth. The large absorption of potassium by flue-cured tobacco resulted the soil Ava-K content continuously decline under long-term continuous cropping^17^.

Previous findings indicated that cropping systems have significantly effects on soil fungal abundance^18,19^. In the present study, we found that the fungal abundance in 5ys was remarkably higher (*P* < 0.01) than that in 3ys and CK (Fig. 1a). The reason for this could be that continuous cropping of the same crop for a long time, has led to the formation of a relatively stable soil environment, and to increase in soil nutrients which promote soil fungi growth. This finding was measured as previously described^20^. In addition, flue-cured tobacco root secretions contain many phenols and acids which provide nutrients for the growth of fungus^21^. Therefore, we concluded that keep cropping flue-cured tobacco increased the soil fungal abundance.

In this study, we found Ascomycota, Basidiomycota and Mortierellomycota were the dominant phyla. Ascomycota was the most abundant in all of the investigated cropping systems (Fig. 2a), consistent with the results of previous studies^22,23^. Ascomycetes are saprotrophic soil fungi which play a major ecological role as decomposers in nature, and influenced by plant species and cropping systems. Continuous cropping reduced soil enzyme activity, and decreased abundance of Ascomycota and Basidiomycota^24^. Mortierellomycota is a distinct phylum which mostly includes saprotrophs in the soil^25,26^. Continuous cropping was significantly correlated with relative abundance of Mortierellomycota. Previous studies have found that Mortierellomycota can dissolve mineral phosphorus in the soil and increase soil nutrient contents by synthesizing and secreting oxalic acid^27,28^. This is consistent with our findings (Fig. 2a, Table 1), where the relative abundance of Mortierellomycota and the Ava-P content in the soil were the hightest in the 5 year cropping system (5ys). At the order level, Sordariales were the dominant order in CK. However, with the increase of continuous cropping years, the relative abundance of Sordariales decreased continuously. Previous studies found that Sordariales have strong ability to decompose lignose and cellulose^29^. Moreover, they can reduce the risk of diseases caused by *Fusarium spinosa* and other pathogens^30^. In the present study, the relative abundances of Hypocreales, Mortierellales, and Mucorales in 5ys were significantly higher than that of 3ys and CK (Fig. 2b). Hypocreales are common in the soil and act as quick decomposers of plant tissue^31,32^. However, many fungi belonging to this order (e. g.: *Fusarium, Cylindrocarpon* etc.) are potential pathogens of flue-cured tobacco. Long-term continuous cropping can accelerates the accumulation of plant autotoxins and increase the risk of of plant diseases^33^. In this study, we found that the difference in fungal community structure between 3ys, 5ys and CK was mainly reflected in Mortierellomycota populations, which was fungal decomposer in soils. We hypothesized that the difference in the abundance of main soil fungal community under different continuous cropping durations might be a consequence of different soil nutrients statuses.

Continuous cropping significantly influenced the soil fungal diversity. Previous studies demonstrated that soil fungal diversity enlargement with the increase in the duration of continuous cropping of Saffron^34^, Sweet Potato^35^, and soybean^36^. In the present study, alpha diversity of fungal communities in 5ys was higher than 3ys and CK (Fig. 1b,c). The main reason for this could involve the chang in soil properties caused by the increase in continuous cropping duration, which is significantly related to the fungal diversity^37^. Continuous cropping of flue-cured tobacco for five yeas result in the formation of a relatively stable soil environment rich in soil nutrients, which was conducive to the increase in fungal community diversity. Two beta diversity methods, NMDS and PCoA, were adopted to compare and analyze taxonomic and phylogenetic measures of fungal community structure in soil under different cropping systems. There was an obvious separation between 3ys, 5ys, and CK, indicating that continuous cropping of flue-cured tobacco had very different influences on soil fungal community structure. Compared with 3ys and CK, 5ys caused significant alter in fungal community composition. This result was consistent with previous research^38,39^. Differences between the cropping systems and associated fungal patterns can be explained by differences in soil physico-chemical properties. The results of the Mantel test showed that TN, Ava-P, Ava-K, and Catalase activity contributed to the changes in fungal community structure of flue-cured tobacco (Table 2). The result of RDA suggested that the Ava-K and Ava-P had the greatest influence in shaping the fungal community structure (Fig. 4). Our findings contrasted with Cleveland et al. and Liu et al., which reported that soil SOC and C/N ratio are the most impotant factors of soil fungal composition^40,41^. However, our findings were consistent with Li and Liu^42^, in which the soil fungal community distribution varied in a “case by case” manner. The most important nutrient for the flue-cured tobacco was found to be Ava-K, a nutrient which has great influence on the growth and development of flue-cured tobacco and the microbial community in the soil^43^. We hypothesized that with the increase in the duration of continuous cropping, the absorption of potassium by flue-cured tobacco plants and dissolution of soil inorganic phosphorus affected the contents of Ava-K and Ava-P in the soil, leading to the changes in fungal community structure in the mountains of southwest China.

Long-term continuous cropping of flue-cured tobacco resulted in a formation of complex co-occurrence fungal network. In present study, the co-occurrence network analysis was used to explor the interaction about fungal species under different cropping systems of flue-cured tobacco. Compared to the cropping for 1 year, the fungal communities under continuous cropping formed more complex and stabilize networks (Fig. 5, Table 3). This finding is consistent with previous study^44^. The soil fungal network in 5ys soil had highest number of nodes and edges than those in 3ys and CK, indicating that long-term continuous cropping had a larger-sized network, and more fungal taxa were involved in potential microbial interactions. This finding is consistent with Huang et al.^45^ and Li^44^. Previous reported that the soil in a long-term continuous cropping system has similar fungal network properties as the soil in a healthy crop rotation system^14^. Understanding the interactions among microbial taxa can allow us to discover potential keystone taxa and identify the members that maintain community co-occurrence patterns^46^. In present study, we observed that the keystone OTUs in the continuous cropping treatments were not those of considered ubiquitous in the soil. This results in accordance with previous studies which found that some low relative abundance taxa may be the pivotal roles in soil fungal community networks^47,48^. In this study, the fungal keystone taxon OTU246 (*Geminibasidium sp.*) obtained from the soils under continuous cropping belongs to a new lineage of xerotolerant and heat-resistant Basidiomycetes. This type of fungus has the ability to decompose and release inorganic phosphorus in the soil^49^. This showed that these keystone species may play essential roles in soil ecosystem because of their beneficial function of increasing the content of available soil nutrients. The keystone taxa OTU65 was annotated with *Cylindrocarpon sp*.. Numerous studies have shown that *Cylindrocarpon* secrete toxins in the soil, and it has been shown that they act as the potential pathogens which can be responsible for many obstacles in continuous cropping of crop^50,51^. Some studies found that keystone taxa play a significant role in the process of soil organic matter decomposition^52^. Therefore, a more detailed study of these types of keystone functional taxa may contribute to the reduction of the harm inposed by soil pathogens in order to improve the soil environment after long-term continuous cropping of flue-cured tobacco.

## Conclusions

Overall, continuous cropping of flue-cured tobacco significantly changed the soil properties and increased the relative abundance and diversity of soil fungal communities. In particular, continuous cropping increased the relative abundance of Mortierellales and Hypocreales. Moreover, long-term continuous cropping had more complex and stabilize network. This study also indicated that differences in soil Ava-K and Ava-P may have important effects on fungal community structure. In summary, our study revealed that long-time continuous cropping of flue-cured tobacco caused a significant influnce on the soil environment, leading to significant variations in soil properties and the abundance and diversity of fungal communities. Future research should focus on the keystone taxa of the co-occurrence fungal network, and explore ways to improve soil environment after long-term continuous cropping of flue-cured tobacco.

## Materials and methods

### Experimental site

This study was performed in a long-term field experimental site at Panzhihua Flued-cure Tobacco Experimental Station, Panzhihua City, Sichuan Province, which is situated the mountainous area of southwest China (27°04′N and 101°45′E, 2080 m a.s.l.). The experimental area belongs to the subtropical dry-hot valley climate. The mean annual precipitation of the area is 1065 mm, and it is mainly concentrated in June to October. The average sunshine duration is 2307 h, and the mean annual temperature is 19.2 °C.

The soils of the investigated area are classified as Alumi-Ferric Alisols according to the FAO-UNESCO Soil Map of the World. The experimental field is flat with uniform fertility. The fertilization scheme used for flue-cured tobacco included annual application of compound fertilizer at the rate of 750 kg ha^−1^ (N:P_2_O_5_:K_2_O = 1:15:3). The variety of flue-cured tobacco used in this experiment was “Yunyan87”, which was provided by the China Tobacco Import and Export Sichuan Co. Ltd.

### Experimental design and soil sampling

Three different treatments were established for this research: (1) flue-cured tobacco continuously cropped for 3 years, (2) continuously cropped for 5 years, and (3) flue-cured tobacco cropping for 1 year, abbreviated as 3 yr, 5 yr and CK, respectively. All treatments were conducted using a randomized block design with three replicates. The plot size was 330 m^2^ (33 m × 10 m). Flue-cured tobacco was transplanted in the experimental field on May 1th solstice August 31th each year. The rest of the time (September 1th solstice April 30th of the following year) no crops were planted and the experimental field was fallow.

On July 5th, 2018 (65 days after transplanting the flue-cured tobacco plants), five soil samples were collected from the 0–20-com soil layer of the the roots of the flue-cured tobacco on random locations in each plot and mixed into a composite sample. Large rocks and roots were removed by sieving at 2 mm in the field just after the sampling. Nine soil samples were collected and analysed in the study (three treatments × three replicates per treatment). The samples were rapidly transported to the laboratory where they were sieved and divided into two portions. One portion was stored at − 80 °C for DNA extraction, and the other was stored at 4 °C for the soil properties and enzyme activity determination.

### Analysis of soil properties

The soil pH was determined as described in Soil Quality-Determination of pH MOD ISO 103,090:2005(E) using a pH meter (CyberScan pH 510, Thermo Fisher Scientific, USA). Soil total N (TN), along with concentrations of ammonium-nitrogen (NH^4+^-N) and nitrate-nitrogen (NO^3−^-N), were determined using the procedure described in Singh et al.^53^ Soil organic carbon (SOC), available phosphorus (Ava-P), and available potassium (Ava-K) in the soil were assayed as described in Zhu et al.^54^ Sucrase, Urease and Catalase activities were determined as described in Wang et al.^55^.

### DNA extraction and quantitative PCR analyses

Total DNA was mixed DNA extracted from 0.5 g fresh soil three times using a FastDNA® SPIN Kit for Soil (MP Biomedicals Co., Ltd., California, USA) according to the manufacturer’s protocols. The concentration of extracted DNA was determined using the NanoDrop 2000 spectrophotometer ((Thermo Scientific, Wilmington, DE, USA), and its quality and integrity was assessed by 1% agarose gels electrophoresis.

The copy numbers of fungi ITS gene was measured by absolutely quantitative PCR (qPCR) using a Biosystems QuantStudio 7 Real-Time PCR System (Life Technologies Inc., USA). The primers ITS1F/ITS2R were used to amplify a 300-bp fragment of the ITS gene from fungi^57^. The qPCR reactions were performed in triplicate 20 μL mixture, containing 10 μL of 2 × TB GreenPremix Ex TaqII (Tli RNaseH Plus), 1.0 μl of each primer(10 μM), 1 μl of BSA, 0.4 μl of ROX Reference Dye II, 4.6 μl of Nuclease-free H_2_O, 2 μl of template DNA (10–50 ng). The qPCR conditions were as follows: 95 °C for 5 min; 40 cycles of 95 °C for 60 s, 51 °C for 60 s, 72 °C for 60 s; and a final melting cycle to produce a melting curve for quality control. All runs had standard efficiency curves of R^2^ > 0.99 and efficiencies of 75.41%.

### Illumina sequencing

The fungal ITS2 region was amplified by PCR using primers 2045F (5′-GCATCGATGAAGAACGCAGC-3′) and 2390R (5′-TCCTCCGCTTATTGATATGC-3′), details of the practice is described as Bellemain et al.^56^ The resultant PCR products were purified, pooled in equimolar concentrations, and paired end sequenced using the NovaSeq platform ((Illumina, Inc., CA, USA) at Realbio Genomics Institute (Shanghai, China). The fungi ITS rRNA gene sequences were deposited into the National Center for Biotechnology Information (NCBI) Sequence Read Archive (SRA) database under the accession number SRP241330.

### Statistical and bioinformatics analysis

The raw fastq data were demultiplexed and quality-filtered by Trimmomatic, and merged by FLASH. The ITS2 tags over a 500-bp sliding window at any site with an average quality score that were lower than 220 were truncated. The average Phred score of the bases was no less than 20 (Q20) and there were no more than three ambiguous N. The Operational Taxonomic Units (OTUs) were assigned at 97% similarity using UPARSE (https://drive5.com/uparse/), and chimeric sequences were identified and removed using Userach (version 7.0.1090). Each representative tag was assigned to a taxon by RDP Classifier against the RDP database (https://rdp.cme.msu.edu/) using confidence threshold of 0.8.

The Shannon’s H value and Simpson’s diversity index were calculated to evaluate the alpha diversity under different cropping system. Estimation of beta diversity and phylogenetic community comparisons were performed using weighted and unweighted UniFrac distance matrices. Non-metric multidimensional scaling (NMDS) was used to illustrate the clustering of the samples. Principal coordinate analysis (PCoA) based on Bray–Curtis distance was performed to test the significance of separation between different cropping systems. Mantel tests were used to determine the correlations between soil properties and fungal community structure. The relationships between soil properties and the OTUs of soil fungi were evaluated using Redundancy analysis (RDA)^57^. Network analyses were visualized with Gephi to reveal the interactions between fungal species^14,58^.

All statistical analyses (analysis of variance and Tukey’s test) as well as Spearman’s rank correlations between abundant phyla and soil properties were performed using the SPSS software package (v19.0, SPSS Inc.). NMDS, PCoA, RDA, and Mantel tests were performed using the “vegan” package of the R v.3.20 statistical environment. Network analysis was performed in the R environment using “WGCNA” package based on Spearman’s rank analysis. A *P* < 0.05 was considered to indicate statistical significance.
